# *N*-Methyl-D-Aspartate Receptor Link to the MAP Kinase Pathway in Cortical and Hippocampal Neurons and Microglia Is Dependent on Calcium Sensors and Is Blocked by α-Synuclein, Tau, and Phospho-Tau in Non-transgenic and Transgenic APP_Sw,Ind_ Mice

**DOI:** 10.3389/fnmol.2018.00273

**Published:** 2018-08-28

**Authors:** Rafael Franco, David Aguinaga, Irene Reyes, Enric I. Canela, Jaume Lillo, Airi Tarutani, Masato Hasegawa, Anna del Ser-Badia, José A. del Rio, Michael R. Kreutz, Carlos A. Saura, Gemma Navarro

**Affiliations:** ^1^Molecular Neurobiology Laboratory, Department of Biochemistry and Molecular Biomedicine, Universitat de Barcelona, Barcelona, Spain; ^2^Centro de Investigación en Red, Enfermedades Neurodegenerativas (CIBERNED), Instituto de Salud Carlos III, Madrid, Spain; ^3^Department of Dementia and Higher Brain Function, Tokyo Metropolitan Institute of Medical Science, Tokyo, Japan; ^4^Department de Bioquímica i Biologia Molecular, Institut de Neurociències, Universitat Autònoma de Barcelona, Bellaterra, Spain; ^5^Molecular and Cellular Neurobiotechnology, Institute of Bioengineering of Catalonia, The Barcelona Institute of Science and Technology, Barcelona, Spain; ^6^Department of Cell Biology, Physiology and Immunology, Universitat de Barcelona, Barcelona, Spain; ^7^RG Neuroplasticity, Leibniz Institute for Neurobiology, Magdeburg, Germany; ^8^Leibniz Group Dendritic Organelles and Synaptic Function, Center for Molecular Neurobiology, ZMNH, University Medical Center Hamburg-Eppendorf, Hamburg, Germany; ^9^Department of Biochemistry and Molecular Biology, Faculty of Pharmacy, Universitat de Barcelona, Barcelona, Spain

**Keywords:** Alzheimer’s disease, calmodulin, calneuron-1, caldendrin, NCS1, extracellular signal-regulated kinase, glutamate receptor, proximity ligation assay

## Abstract

*N*-methyl-D-aspartate receptors (NMDARs) respond to glutamate to allow the influx of calcium ions and the signaling to the mitogen-activated protein kinase (MAPK) cascade. Both MAPK- and Ca^2+^-mediated events are important for both neurotransmission and neural cell function and fate. Using a heterologous expression system, we demonstrate that NMDAR may interact with the EF-hand calcium-binding proteins calmodulin, calneuron-1, and NCS1 but not with caldendrin. NMDARs were present in primary cultures of both neurons and microglia from cortex and hippocampus. Calmodulin in microglia, and calmodulin and NCS1 in neurons, are necessary for NMDA-induced MAP kinase pathway activation. Remarkably, signaling to the MAP kinase pathway was blunted in primary cultures of cortical and hippocampal neurons and microglia from wild-type animals by proteins involved in neurodegenerative diseases: α-synuclein, Tau, and p-Tau. A similar blockade by pathogenic proteins was found using samples from the APP_Sw,Ind_ transgenic Alzheimer’s disease model. Interestingly, a very marked increase in NMDAR–NCS1 complexes was identified in neurons and a marked increase of both NMDAR–NCS1 and NMDAR–CaM complexes was identified in microglia from the transgenic mice. The results show that α-synuclein, Tau, and p-Tau disrupt the signaling of NMDAR to the MAPK pathway and that calcium sensors are important for NMDAR function both in neurons and microglia. Finally, it should be noted that the expression of receptor–calcium sensor complexes, specially those involving NCS1, is altered in neural cells from APP_Sw,Ind_ mouse embryos/pups.

## Introduction

As longevity increases, neurodegenerative diseases such as Alzheimer’s (AD) and Parkinson’s (PD) diseases become a challenge for health and social security systems. In consequence, there is an urgent need for interventions that either prevent neurodegeneration or delay disease progression. Whereas PD management includes successful therapeutic strategies for symptom management, they do not stop neurodegeneration. The situation in the case of AD is worse; the current anti-AD drugs, acetylcholinesterase inhibitors, and *N*-methyl-D-aspartate receptor (NMDAR) modulators are of little anti-symptomatic efficacy and do not prevent disease progression (Gauthier et al., 2016; Szeto and Lewis, 2016).

The NMDAR is one of the most important mediators of excitatory neurotransmission in the brain. It is a tetrameric protein complex formed by two GluN1 and a combination of one or two GluN2A or GluN2B subunits. Receptor subunit expression varies in different brain regions and they convey slightly different neural responses. Interestingly, cortical and hippocampal receptors contain mainly GluN2A and GluN2B subunits (Watanabe et al., 1993; Monyer et al., 1994; Laurie et al., 1997), being those subunits associated to learning and memory (Woodhall et al., 2001; Bidoret et al., 2009). A decrease in NMDAR expression and a variation in subunit composition has been reported in senescence and in AD animal models (Ułas and Cotman, 1997; Wakabayashi et al., 1999; Mishizen-Eberz et al., 2004).

Irrespective of the fact that the NMDAR is a target of current anti-AD medications (memantine), extrasynaptically located NMDA receptors have a relevant role in neurodegeneration. Activation of extrasynaptic receptors may not only regulate expression of Tau (Paterlini et al., 1998) but they also induce transcriptional inactivation of CREB (Rönicke et al., 2011; Grochowska et al., 2017) and also contribute to early synaptic dysfunction. Thus, NMDAR might be relevant targets to prevent early synaptic dysfunction and potentially delay neuronal cell death.

Neural cells express calmodulin, which is ubiquitously expressed, and some other specific calcium-binding proteins that, upon Ca^2+^ binding, participate in events related to neurotransmission and plasticity. One of them, frequenin/NCS1 was first identified in the nervous system of *Drosophila* (see Dason et al., 2012 for review) and later found to be a relevant calcium sensor in the central nervous system of mammals. NCS1 like calneuron-1 and caldendrin, contains EF hand domains that participate in Ca^2+^ binding and mediate the conformational changes that unfolds a myriad of events affecting signaling pathways and impacting on gene transcription (McCue et al., 2010; Burgoyne and Haynes, 2012). Affinity for Ca^2+^ is variable and, for instance, calcium binds with less affinity to NCS1 than to calneuron-1 (Mikhaylova et al., 2006, 2009). Despite Ca^2+^ is the ion transported across NMDAR (see Pankratov and Lalo, 2014; Paoletti et al., 2013 for review), the modulatory role of EF-hand calcium-binding proteins in NMDA receptor function is poorly understood. In this study, we wanted to assess whether NMDAR may directly interact with calcium sensors in neural cells and whether this might affect the coupling of the NMDAR to downstream effectors.

*N*-methyl-D-aspartate receptor-mediated calcium influx is upstream of several intracellular signaling cascades. Interestingly, NMDARs are also expressed in glial cells where their physiological role is not yet fully elucidated. The first aim of this paper was to look for potential interactions between NMDA receptors and calcium-binding proteins. We identified in both neurons and microglia that calcium-binding proteins may interact with NMDAR and we determined how these proteins affect NMDAR-mediated MAP kinase activation. The second aim was to investigate how such NMDAR signaling may be affected by α-synuclein and Tau proteins. Finally, we analyzed whether the results obtained in non-transgenic mice were similar or not to those obtained using cortical and hippocampal neurons and microglia from the APP_Sw,Ind_ transgenic AD mouse model.

## Results

### NMDAR May Interact With Calneuron-1, Calmodulin, and NCS1

**FIGURE 1:**
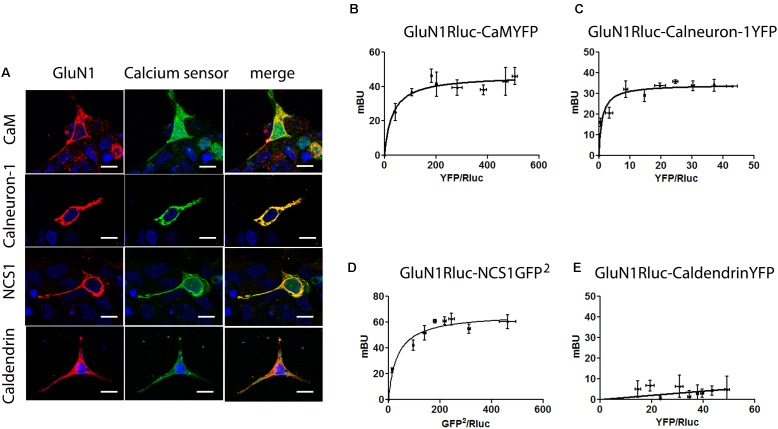
The NMDAR receptor interacts with calmodulin, calneuron-1, and NCS1. **(A)** Confocal microscopy images of HEK-293T cells transfected with cDNAs for GluN1Rluc (0.5 μg), GluN2B (0.3 μg), and CaMYFP (0.5 μg), calneuron-1-YFP (0.75 μg), NCS1YFP (0.5 μg), or caldendrinYFP (0.75 μg). Calcium sensors fused to YFP were identified by their own fluorescence (green) and GluN1Rluc by immunoreactivity (red); co-localization is shown in yellow; scale bars: 10 μm. **(B–E)** BRET [BRET^1^ except BRET^2^ in **(D)**] saturation experiments in HEK-293T cells transfected with cDNAs for GluN1RLuc (0.3 μg), GluN2B (0.3 μg), and increasing amounts of cDNA for CaMYFP (0.05–0.4 μg) **(B)**, calneuron-1YFP (0.05–1 μg) **(C)**, NCS1-GFP^2^ (0.05–0.6 μg) **(D)**, or caldendrinYFP (0.05–1 μg) **(E)**. Values are the mean ± SEM (*n* = 8).

The activation of ionotropic NMDAR results in Ca^2+^-influx and the kinetics as well as amplitude of these synaptic signals are decoded by calcium sensors (Raghuram et al., 2012). Previous data suggest an interaction of NMDAR and CaM (Ehlers et al., 1996) that was here confirmed by means of bioluminescence resonance energy transfer (BRET) assays in a heterologous expression system where GluN1 fused to Rluc and the GluN2B were co-expressed for proper NMDA receptor reconstitution and functional activity (**Figure 1**). First, immunocytofluorescence assays performed in HEK-293T cells co-expressing NMDAR and CaM showed a prominent degree of co-localization. Subsequently, a saturation curve demonstrating a specific interaction was obtained in BRET assays using GluN1Rluc and CaMYFP in the presence (BRET_max_ 47 ± 2 mBU and BRET_50_ 31 ± 6; **Figure 1**) or absence (BRET_max_ 191 ± 8 mBU and BRET_50_ 3.5 ± 1; **Supplementary Figure S1A**) of GluN2. We then tested the interaction of other neuron-specific calcium sensors by immunocytofluorescence and BRET assays. Co-localization was proven for the calcium-binding proteins NCS1 and calneuron-1 but not for caldendrin (**Figure 1A**). BRET results confirmed that NMDAR may interact with NCS1 and calneuron-1 but not with caldendrin. In fact, a saturation BRET curve was obtained using the GluN1Rluc and calneuron-1YFP in the presence (BRET_max_ 34 ± 1 mBU and BRET_50_ 1.0 ± 0.3; **Figure 1C**) or absence (BRET_max_ 43 ± 4 mBU and BRET_50_ 10 ± 3; **Supplementary Figure S1B**) of GluN2. It should be noted that BRET with GluN1Rluc and NCS1-YFP in the presence of GluN2 lead to an unspecific signal. However, in the absence of GluN2, the BRET of the GluN1Rluc and NCS1-YFP pair was saturable (BRET_max_ 73 ± 7 mBU and BRET_50_ 30 ± 7; **Supplementary Figure S1C**). We repeated the energy transfer experiment using BRET^2^ (instead of regular BRET or BRET^1^) and the GluN1Rluc and NCS1-GFP^2^ pair (in the presence of GluN2). BRET^2^ was saturable (BRET_max_ 67 ± 3 mBU and BRET_50_ 35 ± 7; **Figure 1D**). This result can be explained due to a better orientation between donor and acceptor when NCS1-GFP^2^ was used. Finally, in HEK-293T cells expressing GluN1Rluc and caldendrinYFP, the linear unspecific signal observed in the presence (**Figure 1E**) and absence of GluN2 (**Supplementary Figure S1D**) indicates a lack of interaction between this calcium sensor and the NMDAR. In conclusion, these data demonstrate that GluN1/N2-NMDAR may interact with calneuron-1, CaM, and NCS1 but not with caldendrin.

### NMDAR-Mediated ERK1/2 Phosphorylation Is Regulated by CaM, Calneuron-1, and NCS1

**FIGURE 2:**
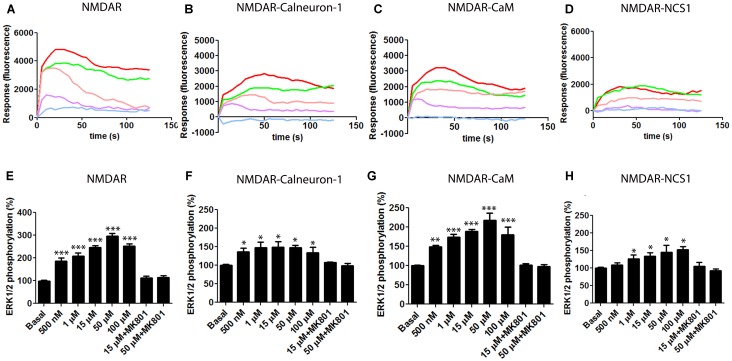
NMDAR -mediated signaling in the presence of calcium sensors. **(A–D)** HEK-293T cells were transfected with the cDNAs for an engineered calcium sensor, 6GCaMP (0.5 μg), GluN1 (0.4 μg), GluN2B (0.25 μg), and calneuron-1 (0.5 μg) **(B)**, CaM (0.3 μg) **(C)**, NCS1 (0.3 μg) **(D)**, or all three **(A)**. Cells were treated with different concentrations of NMDA [500 nM (purple), 1 μM (pink), 15 μM (green), and 50 μM (red)] or pretreated with the NMDAR specific antagonist MK-801 (10 μM) followed by 15 μM NMDA stimulation (blue). Representative traces of intracellular Ca^2+^ responses over time are shown from five independent experiments. **(E,F)** HEK-293T cells were transfected with cDNAs for GluN1 (0.4 μg), GluN2B (0.25 μg), and calneuron-1 (0.5 μg) **(F)**, CaM (0.3 μg) **(G)**, NCS1 (0.3 μg) **(H)**, or all three **(E)**. Cells were treated with different concentrations of NMDA (from 500 nM to 100 μM) or pretreated with the NMDAR specific antagonist MK-801 (10 μM) followed by treatment with NMDA (15 or 50 μM) and ERK1/2 phosphorylation levels were measured. Values are the mean ± SEM (*n* = 7). Significant differences over basal condition were calculated by one-way ANOVA and Bonferroni *post hoc* test (^∗^*p* < 0.05, ^∗∗^*p* < 0.01, and ^∗∗∗^*p* < 0.005).

*N*-methyl-D-aspartate receptor activation leads to the activation of the mitogen-activated protein kinase (MAPK) pathway (Wang et al., 2007). In preliminary experiments, we confirmed that application of NMDA induces the phosphorylation of ERK1/2 and increases the level of intracellular calcium while it did not modify the levels of cAMP (data not shown), which were determined as a control because the NMDAR is not coupled to heterotrimeric G proteins linked to adenylate cyclase. We then tested whether co-expression of NMDAR and calcium sensors affected calcium mobilization. In transiently transfected HEK-293T cells co-expressing the receptor and the calcium-binding proteins, NMDA treatment led to a dose–response increase in the calcium signal (**Figure 2**). NMDA-induced increases in Ca^2+^ levels were obtained when co-transfecting the receptor and CaM, calneuron-1, or NCS1. Whereas the maximal effect was similar when the three sensors were heterologously expressed and when CaM was heterologously expressed, the maximal effect was lower when calneuron-1 or NCS1 were individually expressed; interestingly, the peak signal when NCS1 was expressed occurred at lower NMDA concentrations (**Figure 2**). In all cases, pretreatment with the specific NMDAR antagonist MK-801 (10 μM) followed by 15 μM NMDA treatment, completely abolished NMDA-induced calcium signals (**Figure 2**).

We subsequently analyzed how NMDA treatment of cells leads to ERK1/2 phosphorylation in cell expressing NMDAR and the different calcium sensor proteins. HEK-293T cells expressing GluN1 and GluN2 subunits, and CaM, calneuron-1, and NCS1 responded to NMDA treatment and the effect was blocked by the pretreatment with MK-801 (10 μM) (**Figure 2E**). Again, it was observed that exogenously expressed CaM produced the highest levels of NMDA-induced ERK1/2 phosphorylation. Responses were evident but smaller in cells expressing calneuron-1 or NCS1 (**Figure 2**).

### Interaction of NMDAR and Calcium Sensors in Cultured Neurons and Microglia

**FIGURE 3:**
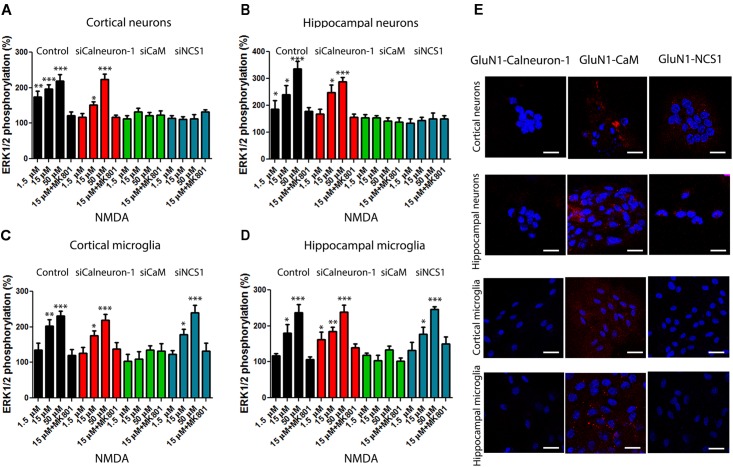
NMDAR-mediated signaling in primary cultures of neurons and microglia from cortex and hippocampus. **(A–D)** MAPK phosphorylation levels were analyzed after stimulating with NMDA (at the indicated concentrations) primary cultures of cortical **(A)**, or hippocampal neurons **(B)**, or of cortex **(C)** or hippocampal microglia **(D)**. Experiments were performed in cells transfected or not (black bars) with siRNA to silence calneuron-1 (red bars), CaM (green bars), or NCS1 (blue bars) expression. Different concentrations of NMDA (from 50 to 1.5 μM) were used to activate the receptor and the NMDAR antagonist, MK-801 (10 μM), was used for specificity checking. Labels in *X* axis are equal in bar graphs of panels **(A–D)**. Values are the mean ± SEM (*n* = 12). Significant differences over basal condition were calculated by one-way ANOVA and Bonferroni *post hoc* test (^∗^*p* < 0.05, ^∗∗^*p* < 0.01, and ^∗∗∗^*p* < 0.005). **(E)** Proximity ligation assays (PLAs) were performed in primary cultures of mice cortex or hippocampus and primary antibodies specific for NMDAR and for calneuron-1, CaM, or NCS1. Confocal microscopy images are shown (superimposed sections) in which heteromers appear as red clusters (in neurons or microglia). Scale bars = 10 μm (neurons) and 20 μm (microglia). In all cases, cell nuclei were stained with Hoechst (blue).

To demonstrate the role of calcium sensor modulation of NMDAR signaling toward the MAP kinase pathway, we moved to primary cultures from mouse brain. When cortical neurons kept for 12 days in culture were treated with increasing concentrations of NMDA (1.5–50 μM), ERK1/2 phosphorylation was obtained (**Figure 3A**). It should be noted that no segregation of synaptic and extrasynaptic NMDAR was yet evident in these cultures. We subsequently used a RNA-based silencing approach to assess the role of each calcium sensor on the NMDAR-mediated responses. When calneuron-1 expression was reduced, the signal obtained was slightly reduced and/or similar depending on the NMDA concentration (**Figure 3A**). By contrast, the effect of NMDA was totally blocked when CaM or NCS1 expressions were silenced (**Figure 3A**). Equivalent results were obtained in neurons from mouse hippocampus (**Figure 3B**). These results in cortical and hippocampal neurons indicate that CaM or NCS1 allows signaling from NMDAR activation to ERK1/2 phosphorylation.

Similar experiments were performed in primary cultures of microglia from cortex or hippocampus. Rather surprisingly, it was observed that NMDAR activation in microglia leads to significant ERK1/2 phosphorylation. In cortical and hippocampal microglia, the NMDAR signal was not blunted when siRNAs against calneuron-1 or NCS1 were used but only when CaM expression was silenced (**Figures 3C,D**). NCS1 is barely expressed in microglia while calneuron-1 (Hradsky et al., 2015) is not expressed. To obtain more information concerning the occurrence of interactions between NMDAR and calcium sensors, *in situ* proximity ligation assays (PLAs) were performed. The technique is suitable to detect complexes of two endogenous proteins, either in tissue slices or in primary cell cultures. PLA showed that cortical and hippocampal neurons display complexes of NMDAR with both CaM and NCS1 sensors (**Figure 3E**). By contrast, in microglial cultures, NMDAR only interacted with CaM (**Figure 3E**). The PLA specificity was demonstrated by lack of signal when a primary antibody was omitted (**Supplementary Figure S2**). These results fit with the results of ERKs phosphorylation, namely, NMDAR interact with and its signaling to MAPK depends on CaM and NCS1 in cortical and hippocampal neurons, whereas the signaling *via* NMDAR in cortical and hippocampal microglia only depends on CaM.

### α-Synuclein Fibrils Block NMDA-Induced MAPK Activation

**FIGURE 4:**
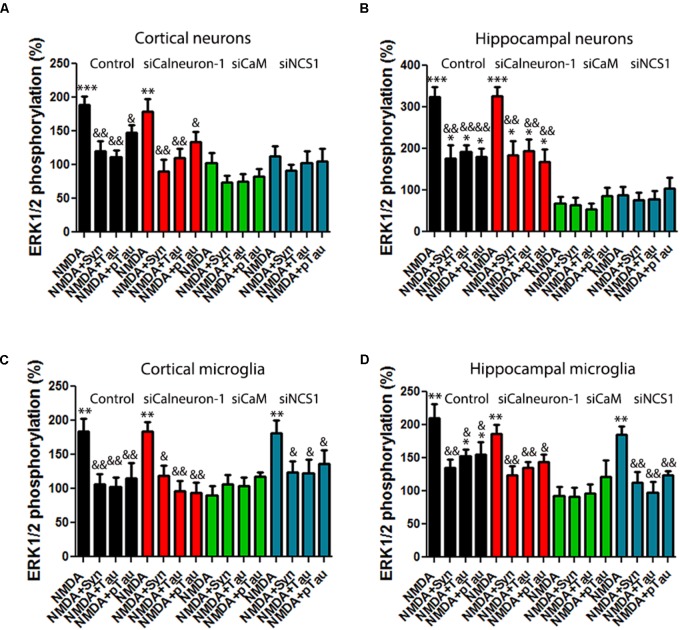
Acute treatment with Tau, p-Tau, or α-synuclein inhibits NMDAR-mediated signaling in primary cultures. **(A–D)** MAPK phosphorylation levels were analyzed after stimulating primary cultures of cortical **(A)**, or hippocampal neurons **(B)**, or of cortical **(C)**, or hippocampal microglia **(D)**. Labels in *X* axis are equal in all bar graphs. Experiments were performed in cells transfected or not (black bars) with siRNA to silence calneuron-1 (red bars), CaM (green bars), or NCS1 (blue bars) expression. Assays were performed in cells treated with α-synuclein, Tau, or p-Tau for 2 h prior to 15 μM NMDA addition. Values are the mean ± SEM (*n* = 10). Significant differences over non-treated cells (^∗∗^*p* < 0.01 and ^∗∗∗^*p* < 0.005) or over NMDA treatment (^&^*p* < 0.05 and ^&&^*p* < 0.01) were calculated by one-way ANOVA and Bonferroni *post hoc* test.

It is well established that α-synuclein is a presynaptic protein that contributes to PD pathogenesis. First, to test the potential effect of recombinant human α-synuclein on NMDAR-mediated activation of the MAPK pathway, primary cultures of hippocampal or cortical neurons were treated with increasing concentrations of α-synuclein fibrils obtained by sonication (1–100 μg/L) (**Supplementary Figures S3D,H**). Thus, in subsequent experiments, the concentration of α-synuclein used was 10 μg/L. In acute treatment, the protein was added 2 h prior treatment with NMDA, whereas in chronic treatment, α-synuclein was preincubated for 7 days. ERK1/2 phosphorylation assays were performed in primary cultures of neurons or microglia from cortex or hippocampus, treated or not with siRNAs able to knockdown calneuron-1, CaM, or NCS1 expression. First of all, we observed that in all neurons and glia (hippocampal and cortical) cells, the acute treatment completely abolished the effect of NMDAR on ERK1/2 phosphorylation (**Figure 4**). When cultures were treated with siRNA for calneuron-1 (red), CaM (green), or NCS1 (blue), the effect was similar. Accordingly, when CaM and NCS1 in neurons or CaM in microglia are silenced thus blocking the NMDA response, α-synuclein could not display any effect. When similar experiments were undertaken under a chronic condition (10 μg/L α-synuclein for 7 days), the results were similar (**Supplementary Figure S4**). Adenosine deaminase (ADA), which binds to various cell surface receptors that are widely expressed in CNS and peripheral cells [CD26/DPPIV and adenosine receptors (Franco et al., 1988; Corset et al., 2000; Ruiz et al., 2000; Herrera et al., 2001; Pacheco et al., 2005)] served as a negative control; ADA did not affect the NMDAR-mediated ERK1/2 phosphorylation (**Supplementary Figure S3**). In summary, acute or chronic α-synuclein fibril treatment of primary cultures blocks engagement of MAP kinase by NMDA.

### Tau and p-Tau Block NMDA-Induced MAPK Activation

Tau is a microtubule-associated protein that interacts with tubulin. Normal adult human brain Tau contains 2–3 moles phosphate/mole of Tau protein (Iqbal et al., 2010) while AD patients contain hyperphosphorylated Tau, which is an aberrant form leading to neurofibrillary tangles. Interestingly, *trans*-synaptic spreading of pathological Tau among interconnected neural circuits have been postulated to be critical in tauopathies and linked to progression of AD pathology (Medina and Avila, 2014; Goedert et al., 2017; Mudher et al., 2017). To elucidate the relevance of Tau and p-Tau effects over NMDAR function, experiments similar to those above described for α-synuclein were performed in primary cultures of cells pre-treated with either Tau of p-Tau. First, neuronal primary cultures were treated with increasing concentrations of Tau (0.05–5 μg/L) and p-Tau (0.05–5 μg/L) (**Supplementary Figures S3B,C,F,G**). In subsequent experiments, the concentration of Tau or p-Tau was 0.5 μg/L. In acute treatment, each of these proteins was added 2 h prior treatment with NMDA, whereas in chronic treatments, cells were incubated for 7 days with Tau or p-Tau. The results showed that both p-Tau and Tau inhibited NMDAR function in acute (**Figure 4**) and also in chronic conditions (**Supplementary Figure S4**). In summary, Tau and p-Tau markedly affected receptor signaling to MAP kinases in both neurons and microglia from cortex or hippocampus. ERK1/2 phosphorylation assays were performed in primary cultures of neurons or microglia from cortex or hippocampus, treated or not with siRNAs able to knockdown calneuron-1, CaM, or NCS1 expression. When CaM and NCS1 in neurons or CaM in microglia were silenced thus blocking the NMDA response, Tau or p-Tau could not display any effect.

### NMDA Receptor/Calcium Sensor Interactions in Neurons From APP_Sw,Ind_ Transgenic Model

**FIGURE 5:**
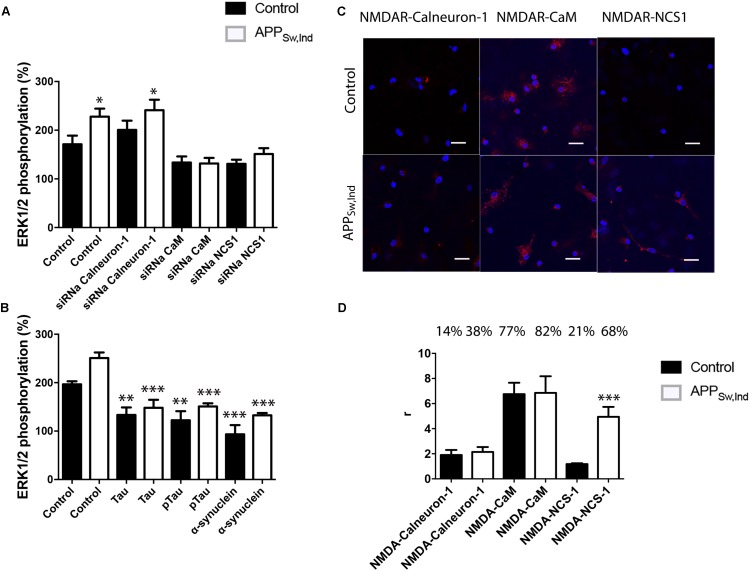
NMDAR-mediated signaling in primary neurons from APP_Sw,Ind_ mice. **(A,B)** Primary cultures from non-transgenic control (black bars) and APP_Sw,Ind_ (white bars) embryos were isolated and transfected or not with siRNA for silencing calneuron-1, CaM, or NCS1. In **(A)**, neurons were treated with 15 μM NMDA, while in **(B)**, neurons were previously treated with α-synuclein, Tau, or p-Tau for 2 h prior to 15 μM NMDA addition; finally, ERK1/2 phosphorylation was measured. Values are the mean ± SEM (*n* = 6). One-way ANOVA followed by Bonferroni’s multiple comparison *post hoc* test were used for statistics analysis. ^∗^*p* < 0.05, ^∗∗^*p* < 0.01, and ^∗∗∗^*p* < 0.001 versus NMDA treatment; comparing only white (non-transgenic data) or only black (transgenic data) bars. **(C)** PLA performed in primary cultures from non-transgenic control (black bars) and APP_Sw,Ind_ (white bars) embryos, using specific primary antibodies raised against NMDAR and calneuron-1, CaM, or NCS1. Confocal microscopy images (stacks of four consecutive planes) show heteroreceptor complexes as red clusters surrounding Hoechst-stained nuclei (blue). Scale bar: 15 μm. The bar graph **(D)** shows the number of red dots/cell and the number above bars indicate the percentage of cells presenting red dots. Values are the mean ± SEM (*n* = 5). One-way ANOVA followed by Bonferroni’s multiple comparison *post hoc* test was used for statistics analysis (^∗∗∗^*p* < 0.001, versus control).

*N*-methyl-D-aspartate receptor function is altered in neurons affected by AD. Accordingly, we isolated primary cultures of neurons from transgenic APP_Sw,Ind_ and control mice to check for occurrence of complexes (by PLA) and for the integrity of the link of NMDAR to the MAPK pathway. First of all, NMDA potentiated ERK1/2 phosphorylation in APP_Sw,Ind_ transgenic mice (white bars) with respect to phosphorylation in control animals (black bars) (**Figure 5A**). Moreover, when neurons from APP_Sw,Ind_ transgenic mice were transfected with siRNA to silence CaM or NCS-1 expression, the MAPK phosphorylation was completely abolished, indicating that CaM and NCS-1 proteins are (as in control animals) required for NMDAR function. By contrast, silencing of calneuron-1 had no effect over NMDA actions. PLAs developed to assess the formation of NMDAR–calcium sensor complexes in primary cultures showed that in control animals (black bars) 77% of neurons express NMDAR–CaM complexes while only 21 and 14% of neurons showed, respectively, NMDAR–NCS1 and NMDAR–calneuron-1 clusters (**Figure 5C,D**). Looking for differences in transgenic animals, the most striking result was the increase in the percentage of cells expressing NCS1–NMDAR complexes (from 21 to 68%) plus the significant increase in the number of clusters per cell (from circa 2 to 5.5; **Figure 5**). These results suggest in the AD mice model that the link NMDAR–MAPK in neurons requires CaM and NCS1 calcium sensor expression, with a more relevant role of NCS1 as the amount of NMDAR–NCS1 heteromers is altered if compared with control animals.

Finally, NMDAR function was assayed in neuronal primary cultures of APP_Sw,Ind_ transgenic mice treated with Tau, p-Tau, or α-synuclein for 2 h prior to 15 μM NMDA addition. The results show that not only Tau and p-Tau proteins but also α-synuclein were able to significantly decrease NMDA-induced MAPK activation (**Figure 5**). These results indicate that neither endogenous Tau nor p-Tau can revert the higher sensitivity of NMDA action in neurons of transgenic mice.

### NMDA Receptor/Calcium Sensor Interactions in Microglia From APP_Sw,Ind_ Transgenic Model

**FIGURE 6:**
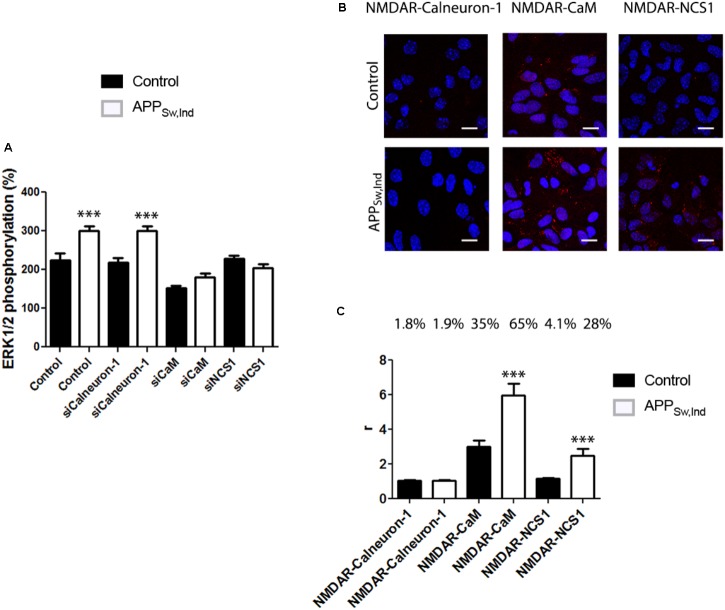
NMDAR-mediated signaling in primary microglia from APP_Sw,Ind_ mice. **(A)** Primary microglial cultures from non-transgenic control (black bars) and APP_Sw,Ind_ (white bars) mice were isolated and transfected or not with siRNA for silencing calneuron-1, CaM, or NCS1. Microglia were treated with 15 μM NMDA and MAPK phosphorylation was measured. Values are the mean ± SEM (*n* = 6). One-way ANOVA followed by Bonferroni’s multiple comparison *post hoc* test were used for statistics analysis (^∗∗∗^*p* < 0.001 versus control). **(B)** Primary microglial cultures from non-transgenic control (black bars) and APP_Sw,Ind_ (white bars) mice were assayed for potential interactions between NMDAR and calcium sensors. PLA was performed using primary cultures of microglia and specific primary antibodies raised against NMDAR and calneuron-1, CaM, or NCS1. Confocal microscopy images (stacks of four consecutive planes) show heteroreceptor complexes as red clusters surrounding Hoechst-stained nuclei (blue). Scale bar: 10 μm. The bar graph **(C)** shows the number of red dots/cell and the number above bars indicate the percentage of cells presenting red dots. Values are the mean ± SEM (*n* = 6). One-way ANOVA followed by Bonferroni’s multiple comparison *post hoc* test was used for statistics analysis (^∗∗∗^*p* < 0.001, comparing each condition versus control, i.e., comparing one by one white versus the corresponding black bar).

To analyze the status of the NMDAR-MAP kinase link in an AD model, primary cultures of microglia from non-transgenic (control) or APP_Sw,Ind_ transgenic mice were cultured and treated with NMDA (15 μM). Interestingly, the results of ERK1/2 phosphorylation indicated that NMDAR function was potentiated in microglia from the APP_Sw,Ind_ transgenic mice (white bars in **Figure 6**). When cultures were transfected with siRNA to silence calneuron-1 expression, a similar result was obtained thus suggesting that calneuron-1 is not involved in the NMDA effect on MAPK activation (**Figure 6A**, microglia from control in black, from transgenic in white). By contrast, when cultures were transfected with siRNA to silence CaM expression, it was observed that the NMDAR signaling completely disappeared in microglia from both control and APP_Sw,Ind_ mice, indicating that CaM interaction with NMDAR is required for receptor-mediated engagement of the MAPK pathway. In fact, these results are in agreement with the data shown in **Figure 3**. Finally, when microglia primary cultures of APP_Sw,Ind_ transgenic mice and control animals were treated with siRNA to decrease NCS1 expression, the abolishment of NMDA effect was only observed in the APP_Sw,Ind_-derived cells (**Figure 6A**). The results indicate that the NCS1–NMDAR interaction is relevant for receptor function in the microglia of the AD mouse model.

Finally, formation of complexes of NMDA receptors and calcium-binding proteins was detected by PLA in primary cultures of microglia from APP_Sw,Ind_ and control mice. Interestingly, we observed that in control animals (black bars), only CaM formed heteromeric complexes with the NMDA receptor (35% of cells showing around three red dots/ cell containing dots) with negligible values for interactions with calneuron-1 or NCS1 (respectively, 1.85 and 4.8% of cells expressing red dots). When APP_Sw,Ind_ microglia was analyzed, the number of complexes between NMDAR and CaM was markedly higher (65% of cells showing around 6 red dots/ cell containing dots), whereas the degree of interaction with calneuron-1 was negligible (1.9% of cells containing red dots). It should be also noted that complexes of NCS1 and NMDAR were also noticeable in the APP_Sw,Ind_ microglia thus indicating a NMDAR-NCS1 interaction in microglia that deserves attention when addressing AD pathophysiology. While the NCS1 expression in microglia from wild-type animals is reportedly low (Averill et al., 2004; Hradsky et al., 2015; Nakamura et al., 2017), the substantial increase in NMDAR-NCS1 complexes shown in **Figures 6B,C**, suggests that expression of NCS1 in microglia from the APP_Sw,Ind_ mice is significant.

## Discussion

The arguably two most relevant neuronal second messengers whose intracellular concentration changes upon cell surface receptor activation are cAMP and Ca^2+^. In our efforts to look for cAMP- and calcium-signaling cross-talk, we have shown direct interactions between calcium sensors and receptors for neurotransmitters/neuromodulators that are coupled to G proteins (GPCRs), i.e., metabotropic receptors. Interestingly, the interaction with calcium sensors in conditions of elevated Ca^2+^ levels results in marked modulation of the signaling events mediated by the cell surface GPCRs expressed in neurons (Lian et al., 2011; Mikhaylova et al., 2011; Navarro et al., 2012a,b, 2014).

In a heterologous expression system, we found that with the exception of caldendrin, all investigated calcium sensors may interact with the GluN1 subunit of NMDAR. Caldendrin is highly abundant in pyramidal neurons (Seidenbecher et al., 1998; Mikhaylova et al., 2018) and also part of the protein complex formed by the calcium sensor and an interacting partner, Jacob, that links NMDAR-mediated signaling to the nucleus (Dieterich et al., 2008) in a cytocrin fashion (Navarro et al., 2017). Our data using a heterologous expression system indicate that no direct interaction occurs with the GluN1 subunit under these conditions.

The NMDAR is one of the upstream players that impact on the MAP kinase signaling pathway. The present study suggests that the coupling of the MAPK pathway to NMDAR activation depends on the expression of calcium sensors. Interestingly, CaM is providing the link in primary cultures of cortical and hippocampal microglia, whereas CaM or NCS1 provide the link in cortical and hippocampal neurons. Among the few studies linking calcium sensors to ionotropic related neurophysiological events, those performed by Jo et al. (2008) in synapses of the perirhinal cortex showed that long-term depression mediated by ionotropic glutamate NMDA receptors involves CaM, whereas long-term depression mediated by metabotropic G-protein-coupled glutamate receptors involves NCS1. In primary cultures of neurons, the link to MAP kinase activation was blocked by α-synuclein, Tau, and p-Tau, which are relevant players in the pathophysiology of proteinopathies such as AD and PD. The effect was similar in neurons and in microglia. Taking into account the similar blockade (both in acute and chronic treatments) by the three different proteins, and the fact that the calcium sensors modulate the coupling of NMDAR to the MAPK pathway, the blockade may result from direct interaction with NMDAR or by interference of signaling events downstream of the receptor.

Microglia, first considered as detrimental when activated due to brain damage or neurodegeneration, appear now with huge potential for neuroprotection (Franco and Fernández-Suárez, 2015). In a previous work using the APP_Sw,Ind_ transgenic AD mice, we found that primary cultures of microglia from pup brain display an activated phenotype (Navarro et al., 2018). As young APP_Sw,Ind_ animals do not display any cognitive impairment, such finding could indicate that activated microglia are neuroprotective in this animal model. Accordingly, we wanted to assess possible alterations in the functioning of NMDAR in such cells. On the one hand, NMDAR are coupled to MAP kinases but with a differential finding respect to that in cells from control animals. In fact, the NMDA-induced increase of ERK1/2 phosphorylation was independent of calneuron-1 but modulated by CaM and NCS1. In parallel assays performed in cells from control animals, the effect was only inhibited by silencing CaM in microglia (or by silencing CaM and NCS-1 in neurons). Further data on assessing the complexes involving NMDA receptors confirmed a significant increase in the number of microglial cells expressing NMDAR/CaM and NMDA/NCS1 complexes and in the number of clusters per cell. In neurons, the relevant increase in both percentage of expressing cells and amount of clusters/cell concerned NMDAR/NCS1 complexes.

The results obtained in primary cultures (neurons and microglia) from transgenic mice show exacerbation of NMDA-induced MAPK activation. These results combined with the effect of pathogenic proteins leads to two possibilities, namely, (i) hyperactivation is independent of the presence of those (endogenously expressed) proteins or (ii) neural cells counteract the effect of α-synuclein fibrils, Tau, and p-Tau by increasing NMDAR signaling function. Accordingly, the molecular underpinnings and potential differences thereof in the relationship between NMDAR and calcium-binding proteins deserves further experimental effort. What it is however relevant in our findings in the AD model is the increase in the expression of NMDAR–NCS1 complexes; the consequences of such significant change (in neurons: > 3-fold in number of cells expressing complexes and >fourfold the number of complexes per cell) deserves a close scrutiny to assess its relevance in AD pathophysiology.

## Materials and Methods

### Reagents

*N*-methyl-D-aspartate and MK-801 were purchased from Tocris Bioscience (Bristol, United Kingdom). Recombinant human α-synuclein was prepared as described (Matsuda et al., 1990) and Tau and p-Tau proteins were kindly provided by Prof. J. Avila (CBM, UAM-CSIC, Madrid, Spain). Detailed descriptions of the elaboration and processing of proteins can be found elsewhere (Pérez et al., 2002; Tarutani et al., 2016).

### Expression Vectors

cDNA for the human version of the GluN1 subunit of NMDAR lacking the stop codon was obtained by PCR and subcloned to RLuc-containing vector (p*RLuc*-N1; PerkinElmer, Wellesley, MA, United States) using sense and antisense primers harboring unique restriction sites for HindIII and BamHI; the generated cDNA encodes a GluN1Rluc fusion protein. cDNA for the human version of GluN2B subunit of NMDAR was subcloned in pcDNA3.1. In functional assays, both cDNAs encoding for GluN1 and GluN2B were cotransfected. CaM gene sequence from pcDNA3 was subcloned in pEYFP-N1 vector (pEYFP: enhanced yellow variant of GFP; Clontech, Heidelberg, Germany), as previously described (Navarro et al., 2009) to generate a plasmid encoding CaM-YFP fusion protein. cDNA constructs encoding NCS1, calneuron-1, or caldendrin in pcDNA3 vectors were subcloned in pEYFP-N1 or pGFP^2^-N1 vectors as previously described in (Navarro et al., 2012b) to generate plasmids encoding NCS1YFP, NCS1GFP^2^, calneuron-1YFP, and caldendrinYFP fusion proteins. The cDNA for calneuron-1, caldendrin, and NCS1, cloned into pcDNA3.1, were amplified (omitting stop codons) using sense and antisense primers harboring unique HindIII and BamHI sites to clone the amplified fragments to be in frame in the pEYFP-N1 or pGFP^2^-N1 vectors.

### APP_Sw,Ind_ Transgenic Mice

APP_Sw,Ind_ transgenic mice (line J9; C57BL/6 background) expressing human APP695 harboring the FAD-linked Swedish (K670N/M671L) and Indiana (V717F) mutations under the PDGFβ promoter were obtained by crossing APP_Sw,Ind_ to non-transgenic (control) mice (Mucke et al., 2000). Control and APP_Sw,Ind_ embryos (E16.5) were genotyped individually and used for microglia cultures as described elsewhere (Navarro et al., 2018). Animal care and experimental procedures were in accordance with European and Spanish regulations (86/609/CEE; RD1201/2005). Mice were handled, as per law, by personnel with the *ad hoc* certificate (issued by the *Generalitat de Catalunya*) that allows animal handling for research purposes.

### Cell Culture and Transient Transfection

HEK-293T cells were grown in in Dulbecco’s modified Eagle’s medium (DMEM) supplemented with 2 mM L-glutamine, 100 U/ml penicillin/streptomycin, and 5% (v/v) heat inactivated fetal bovine serum (FBS) (Invitrogen, Paisley, Scotland, United Kingdom). Cells were maintained in a humid atmosphere of 5% CO_2_ at 37°C. Cells were transiently transfected with the polyethylenimine (PEI, Sigma, St. Louis, MO, United States) method. To prepare mice cortex and hippocampus primary microglial cultures, brain was removed from C57/BL6 mice between 2- and 4-days-old. Microglia cells were isolated as described in (Newell et al., 2015) and plated at confluence of 40,000 cells/0.32 cm^2^ and grown in DMEM medium supplemented with 2 mM L-glutamine, 100 U/ml penicillin/streptomycin, and 5% (v/v) heat inactivated FBS (Invitrogen, Paisley, Scotland, United Kingdom) for 12 days. Neuronal primary cultures were prepared from cortex and hippocampus of fetuses from C57/BL6 pregnant mice. Neurons were isolated as described in Hradsky et al. (2013) and plated at a confluence of 40,000 cells/0.32 cm^2^. Striatal cells were grown in neurobasal medium supplemented with 2 mM L-glutamine, 100 U/ml penicillin/streptomycin, and 2% (v/v) B27 supplement (Gibco) in a 96-well plate for 12 days.

Silencing of constitutively expressed calcium-binding proteins was performed as described elsewhere (Navarro et al., 2014). Briefly, cortical or hippocampal cultures (neurons or microglia) growing in 96-well plates were transfected with the Lipofectamine^®^2000 (Thermo Fisher) method to silence the NCS1, CaM, or calneuron-1 expression using pSuper-NCS-1 vector, pSuper-CaM vector, and calneuron-1 shRNAII (1xconstruct #2; genecopoeia). Cells were incubated for 6–8 h with the cDNA and Lipofectamine^®^ in serum-starved medium. After 6–8 h, the medium was replaced with a complete culture medium. Validation was performed by Western blotting and the reduction in expression from control values (>75% in neurons; >85% in microglia). Rescuing assays were performed in primary cultures (already expressing endogenous CaM) by first silencing CaM using a specific siRNA and further transfection with a vector containing the CaM sequence. The results related to the NMDAR–MAPK link disappeared upon silencing but re-appeared upon “reexpression” of CaM by transfection with the specific cDNA (**Supplementary Figure S5**).

### Preparation of Human α-Synuclein Fibrils

α-Synuclein fibrils were prepared by shaking purified recombinant α-synuclein as described (Masuda-Suzukake et al., 2014; Tarutani et al., 2016). Briefly, purified recombinant α-synuclein (5 mg/ml) containing 30 mM Tris–HCl (pH 7.5), 10 mM DTT, and 0.1% sodium azide were incubated for 7 days at 37°C in a horizontal shaker at 200 rpm, then ultracentrifuged at 113,000 × *g* for 20 min at 25°C. The pellets were washed with saline and ultracentrifuged as before. The resulting pellets were collected as α-synuclein fibrils and resuspended in 30 mM Tris–HCl (pH 7.5). The fibrils were fragmented using a cup horn sonicator (Sonifier^®^ SFX, Branson) at 35% power for 180 s (total 240 s, 30 s on, 10 s off) (Tarutani et al., 2016, 2018). Before use aliquots were left at room temperature and placed in PBS 1× (pH 7.2) to a final concentration of 0.1 μg/μL. These preparations were subjected to 60 pulses of sonication (runtime 30 s: 0.5 s on, 0.5 s off in a BBR03031311digital SONIFIER sonicator). Sonicated fibril preparations were diluted in pre-warmed medium and immediately added to cells.

### Bioluminescence Resonance Energy Transfer (BRET) Assays

For BRET^1^, HEK-293T cells were transiently co-transfected with a constant amount of cDNA encoding for GluN1-RLuc and GluN2B in pcDNA3.1 and with increasing amounts of cDNA corresponding to calneuron-1-YFP, caldendrin-YFP, CaM-YFP, or NCS1-YFP. 48 h after transfection cells were adjusted to 20 μg of protein using a Bradford assay kit (Bio-Rad, Munich, Germany) using bovine serum albumin for standardization. To quantify protein-YFP expression, fluorescence was read in a Mithras LB 940 equipped with a high-energy xenon flash lamp, using a 30-nm bandwidth excitation filter at 485 nm. For BRET measurements, readings were collected 30 s after the addition of 5 μM coelenterazine H (Molecular Probes, Eugene, OR, United States) using a Mithras LB 940, which allows the integration of the signals detected in the short-wavelength filter at 485 nm and the long-wavelength filter at 530 nm. To quantify protein-RLuc expression, luminescence readings were performed 10 min after 5 μM coelenterazine H addition using a Mithras LB 940. For BRET^2^, HEK-293T cells were transiently co-transfected with a constant amount of cDNA encoding for GluN1-RLuc and GluN2B in pcDNA3.1 and with increasing amounts of cDNA corresponding to NCS1-GFP^2^. 48 h after transfection cells were adjusted to 20 μg of protein using a Bradford assay kit (Bio-Rad, Munich, Germany) using bovine serum albumin for standardization. To quantify protein-GFP^2^ expression, fluorescence was read in a Fluostar Optima fluorimeter equipped with a high-energy xenon flash lamp, using a 10-nm bandwidth excitation filter at 405 nm. For BRET measurements, readings were collected 1 min after the addition of 5 μM Deep Blue C (Molecular Probes, Eugene, OR, United States) using a Mithras LB 940, which allows the integration of the signals detected in the short-wavelength filter at 405 nm and the long-wavelength filter at 510 nm. To quantify protein-RLuc expression, luminescence readings were performed 10 min after 5 μM coelenterazine H addition using a Mithras LB 940. The net BRET is defined as [(long-wavelength emission)/(short-wavelength emission)] - Cf, where Cf corresponds to [(long-wavelength emission)/(short-wavelength emission)] for the donor construct expressed alone in the same experiment. GraphPad Prism software (San Diego, CA, United States) was used to fit data. BRET is expressed as milli BRET units, mBU (net BRET × 1,000) (Canals et al., 2003, 2004; Hinz et al., 2018).

### Immunocytofluorescence

HEK-293T cells were transfected with GluN1-RLuc, GluN2B, and calneuron-1-YFP, caldendrin-YFP, CaM-YFP, or NCS1-YFP were fixed in 4% paraformaldehyde for 15 min and washed twice with PBS containing 20 mM glycine before permeabilization with PBS-glycine containing 0.2% Triton X-100 (5 min incubation). HEK-293T cells were treated for 1 h with PBS containing 1% bovine serum albumin and labeled with the primary mouse anti-RLuc antibody, and subsequently treated with: Cy3 anti-rabbit [1/200; Jackson ImmunoResearch (red)] secondary antibodies for 1 h. The YFP-fusion proteins were detected by YFP own fluorescence. Samples were washed several times and mounted with 30% Mowiol (Calbiochem). Samples were observed in a Leica SP2 confocal microscope (Leica Microsystems). Scale bar: 10 μm for neurons and 20 μm for microglia cells.

### Calcium Release

HEK-293T cells were co-transfected with the cDNA for the indicated receptors and 0.75 μg of GCaMP6 calcium sensor (Chen et al., 2013) using PEI protocol (Section “Cell Culture and Transient Transfection”). Forty-eight hours after transfection, cells (150,000 HEK-293T cells/well in 96-well black, clear bottom microtiter plates) were incubated with Mg^2+^-free Locke’s buffer pH 7.4 (154 mM NaCl, 5.6 mM KCl, 3.6 mM NaHCO_3_, 2.3 mM CaCl_2_, 5.6 mM glucose, and 5 mM HEPES) supplemented with 10 μM glycine and receptor ligands were added just a few seconds before readings. Fluorescence emission intensity of GCaMP6 was recorded at 515 nm upon excitation at 488 nm on the EnSpire^®^ Multimode Plate Reader for 335 s every 15 s and 100 flashes per well.

### ERK Phosphorylation Assays

To determine ERK1/2 phosphorylation, HEK-293T cells expressing GluN1 and GluN2B subunits and calneuron-1, CaM, or NCS1, primary cultures of cortex or hippocampus microglia cells or primary cultures of cortex or hippocampus neurons were plated at a density of 40,000 cells/well in transparent Deltalab 96-well microplates and kept at the incubator between 1 and 7 days. Two to four hours before the experiment, the medium was substituted by serum-starved DMEM medium. Then, cells were pre-treated or not for 2 h or 7 days with α-synuclein, Tau, and p-Tau proteins at 37°C followed by treatment at 25°C for 10 min with vehicle or antagonists (MK-801) in serum-starved DMEM medium and stimulated for an additional 7 min with NMDA. Cells were then washed twice with cold PBS before addition of lysis buffer (20 min treatment). Ten microliters of each supernatant were placed in white ProxiPlate 384-well microplates and ERK 1/2 phosphorylation was determined using AlphaScreen^®^SureFire^®^ kit (PerkinElmer) following the instructions of the supplier and using an EnSpire^®^ Multimode Plate Reader (PerkinElmer, Waltham, MA, United States).

### Proximity Ligation Assays (PLAs)

Interactions between NMDAR and calcium sensors were detected using the Duolink II *in situ* PLA detection Kit (OLink; Bioscience, Uppsala, Sweden) following the instructions of the supplier. Primary cultures of neurons and microglia cells were grown on glass coverslips and were fixed in 4% paraformaldehyde for 15 min, washed with PBS containing 20 mM glycine to quench the aldehyde groups, permeabilized with the same buffer containing 0.05% Triton X-100 for 5 min, and successively washed with PBS. After 1 h incubation at 37°C with the blocking solution in a pre-heated humidity chamber, primary cultures were incubated overnight in the antibody diluent medium with a mixture of equal amounts of rat monoclonal anti-NMDAR antibody (1:200, Millipore) and a polyclonal rabbit anti-NCS1 antibody (1:100, Millipore) to detect NMDAR–NCS1 complexes, or and the rabbit polyclonal anti-calneuron-1 antibody (1:100, Abcam) to detect NMDAR-calneuron-1 complexes or and a monoclonal rabbit anti-CaM antibody (1:50, Abcam) to detect NMDAR–CaM complexes. Cells were processed using the PLA probes detecting primary antibodies (Duolink II PLA probe plus and Duolink II PLA probe minus) diluted in the antibody diluent (1:5). Ligation and amplification were done as indicated by the supplier and cells were mounted using the mounting medium with Hoechst (1/200; Sigma). Samples were observed in a Leica SP2 confocal microscope (Leica Microsystems, Mannheim, Germany) equipped with an apochromatic 63× oil immersion objective (N.A. 1.4), and a 405-nm and a 561-nm laser lines. For each field of view, a stack of two channels (one per staining) and four to eight Z stacks with a step size of 1 μm were acquired. A quantification of cells containing one or more red spots versus total cells (blue nucleus) and, in cells containing spots, the ratio *r* (number of red spots/cell), were determined. One-way ANOVA followed by Dunnett’s *post hoc* multiple comparison test was used to compare the values (% of positive cells or *r* spots/cell) obtained for each pair of receptors.

## Author Contributions

GN and RF conceived and designed the experiments. MK, AT, MH, and JdR provided key reagents. CS provided transgenic animals and controls. GN, DA, JdR, JL, AdS-B, IR, and EC performed the experiments and analyzed the data. RF, GN, MK, EC, and CS wrote the paper. All authors have edited the manuscript and received copy of the submitted version.

## Conflict of Interest Statement

The authors declare that the research was conducted in the absence of any commercial or financial relationships that could be construed as a potential conflict of interest.

## Supplementary Material

FIGURE S1 The N1 subunit of NMDAR receptor may interact with calmodulin, calneuron-1 and NCS1. **(A–D)** BRET saturation experiments in HEK-293T cells transfected with cDNAs for GluN1Rluc (0.3 μg), and increasing amounts of cDNA for CaMYFP (0.05–0.4 μg) **(A)**, calneuron-1YFP (0.05–1 μg) **(B)**, NCS1YFP (0.05–0.6 μg) **(C)**, or caldendrin YFP (0.05–1 μg) **(D)**. Values are the mean ± SEM (*n* = 6).

FIGURE S2 Proximity ligation assay (PLA) negative controls for PLAs in primary cultures of neurons and microglia. PLAs were performed in cortical (**A** neurons, **C** microglia) and hippocampal (**B** neurons, **D** microglia) cells incubated with the anti-CaM antibody but omitting the anti-NMDAR antibody. Confocal microscopy images are shown (superimposed sections) in which heteromers appear as red clusters (in neurons or microglia). Scale bars = 10 μm (neurons) and 20 μm (microglia). In all cases, cell nuclei were stained with Hoechst (blue).

FIGURE S3 Tau, p-Tau, and α-synuclein dose–response curves in neuronal primary cultures. **(A–H)** MAPK phosphorylation levels were analyzed in primary cultures of cortical **(A–D)**, or hippocampal neurons **(E–H)**. Assays were performed in cells treated with increasing concentrations of adenosine deaminase (ADA) (5–500 μM). **(A,E)** Tau (0.05–5 μg/L) **(B,F)**, p-Tau (0.05–5 μg/L) **(C,G)**, or α-synuclein (1 μM–100 μg/L) **(D,H)** for 2 h prior to 15 μM NMDA stimulation. Values are the mean ± SEM (*n* = 6). Significant differences over NMDA treatment (control condition) (^∗^*p* < 0.05, ^∗∗^*p* < 0.01, and ^∗∗∗^*p* < 0.005) were calculated by one-way ANOVA and Bonferroni *post hoc* test.

FIGURE S4 Chronic treatment with Tau, p-Tau, or α-synuclein inhibits NMDAR-mediated signaling in primary cultures. **(A–D)** MAPK phosphorylation levels were analyzed after stimulating primary cultures of cortical **(A)**, or hippocampal neurons **(B)**, or of cortex **(C)**, or hippocampal microglia **(D)**. Experiments were performed in cells transfected or not (black bars) with siRNA to silence calneuron-1 (red bars), CaM (green bars), or NCS1 (blue bars) expression. Assays were performed in cells treated with α-synuclein, Tau or p-Tau for 7 days prior to 15 μM NMDA addition. Labels in *X* axis are equal in all bar graphs. Values are the mean ± SEM (*n* = 10). Significant differences over non-treated cells (^∗∗^*p* < 0.01, ^∗∗∗^*p* < 0.005) or over NMDA treatment (^&^*p* < 0.05, ^&&^*p* < 0.01, and ^&&&^*p* < 0.001) were calculated by one-way ANOVA and Bonferroni *post hoc* test.

FIGURE S5 Rescue of (endogenous) CaM silencing upon transfection with the cDNA for CaM. **(A,B)** MAPK phosphorylation levels were analyzed after stimulating primary cultures of cortical neurons **(A)** or cortical microglia **(B)** with 15 μM NMDA. Experiments were performed in cells transfected or not with siRNA to silence CaM; 24 h later, cells were transfected with cDNA for CaM or with the (empty) pcDNA3.1 vector. Experiments in untransfected (left), in siRNA (center), and in siRNA plus CaM (right) cells were preformed simultaneously. Data are the mean ± SEM (*n* = 5). One-way ANOVA followed by Bonferroni’s multiple comparison *post hoc* test were used for statistics analysis (^∗^*p* < 0.05, ^∗∗^*p* < 0.01 versus NMDA treatment).

## References

[B1] Averill S. Robson L. G. Jeromin A. Priestley J. V. (2004). Neuronal calcium sensor-1 is expressed by dorsal root ganglion cells, is axonally transported to central and peripheral terminals, and is concentrated at nodes. *Neuroscience* 123 419–427. 10.1016/j.neuroscience.2003.09.031 14698749

[B2] Bidoret C. Ayon A. Barbour B. Casado M. (2009). Presynaptic NR2A-containing NMDA receptors implement a high-pass filter synaptic plasticity rule. *Proc. Natl. Acad. Sci. U.S.A.* 106 14126–31. 10.1073/pnas.0904284106 19666514 PMC2729031

[B3] Burgoyne R. D. Haynes L. P. (2012). Understanding the physiological roles of the neuronal calcium sensor proteins. *Mol. Brain* 5 1–11. 10.1186/1756-6606-5-2 22269068 PMC3271974

[B4] Canals M. Burgueño J. Marcellino D. Cabello N. Canela E. I. Mallol J. (2004). Homodimerization of adenosine A2A receptors: Qualitative and quantitative assessment by fluorescence and bioluminescence energy transfer. *J. Neurochem.* 88 726–734. 10.1046/j.1471-4159.2003.02200.x 14720222

[B5] Canals M. Marcellino D. Fanelli F. Ciruela F. De Benedetti P. Goldberg S. R. (2003). Adenosine A2A-dopamine D2 receptor-receptor heteromerization: qualitative and quantitative assessment by fluorescence and bioluminescence energy transfer. *J. Biol. Chem.* 278 46741–46749. 10.1074/jbc.M306451200 12933819

[B6] Chen T.-W. Wardill T. J. Sun Y. Pulver S. R. Renninger S. L. Baohan A. (2013). Ultrasensitive fluorescent proteins for imaging neuronal activity. *Nature* 499 295–300. 10.1038/nature12354 23868258 PMC3777791

[B7] Corset V. Nguyen-Ba-Charvet K. T. Forcet C. Moyse E. Chédotal A. Mehlen P. (2000). Netrin-1-mediated axon outgrowth and cAMP production requires interaction with adenosine A2b receptor. *Nature* 407 747–750. 10.1038/35037600 11048721

[B8] Dason J. S. Romero-Pozuelo J. Atwood H. L. Ferrús A. (2012). Multiple roles for frequenin/NCS-1 in synaptic function and development. *Mol. Neurobiol.* 45 388–402. 10.1007/s12035-012-8250-4 22396213

[B9] Dieterich D. C. Karpova A. Mikhaylova M. Zdobnova I. König I. Landwehr M. (2008). Caldendrin-Jacob: a protein liaison that couples NMDA receptor signalling to the nucleus. *PLoS Biol.* 6:e34. 10.1371/journal.pbio.0060034 18303947 PMC2253627

[B10] Ehlers M. D. Zhang S. Bernhardt J. P. Huganir R. L. (1996). Inactivation of NMDA receptors by direct interaction of calmodulin with the NR1 subunit. *Cell* 84 745–755. 10.1016/S0092-8674(00)81052-1 8625412

[B11] Franco R. Fernández-Suárez D. (2015). Alternatively activated microglia and macrophages in the central nervous system. *Prog. Neurobiol.* 131 65–86. 10.1016/j.pneurobio.2015.05.003 26067058

[B12] Franco R. Centelles J. J. Canela E. I. (1988). Determination of the characteristics, properties and homogeneity of rat brain microsomes. *Binding of lactate dehydrogenase, malate dehydrogenase and* 5′ nucleotidase to microsomal membranes. *Biochem. Int.* 16 689–699. 10.1016/j.pneurobio.2015.05.003 2839190

[B13] Gauthier S. Albert M. Fox N. Goedert M. Kivipelto M. Mestre-Ferrandiz J. (2016). Why has therapy development for dementia failed in the last two decades? *Alzheimer’s Dement.* 12 60–64. 10.1016/j.jalz.2015.12.003 26710325

[B14] Goedert M. Eisenberg D. S. Crowther R. A. (2017). Propagation of tau aggregates and neurodegeneration. *Annu. Rev. Neurosci.* 40 189–210. 10.1146/annurev-neuro-072116-031153 28772101

[B15] Grochowska K. M. Yuanxiang P. Bär J. Raman R. Brugal G. Sahu G. (2017). Posttranslational modification impact on the mechanism by which amyloid-β induces synaptic dysfunction. *EMBO Rep.* 18 962–981. 10.15252/embr.201643519 28420656 PMC5452034

[B16] Herrera C. Casadó V. Ciruela F. Schofield P. Mallol J. Lluis C. (2001). Adenosine A2B receptors behave as an alternative anchoring protein for cell surface adenosine deaminase in lymphocytes and cultured cells. *Mol. Pharmacol.* 59 127–134. 10.1124/mol.59.1.127 11125033

[B17] Hinz S. Navarro G. Borroto-Escuela D. Seibt B. F. Ammon C. Filippo De E. (2018). Adenosine A2A receptor ligand recognition and signaling is blocked by A2B receptors. *Oncotarget* 9 13593–13611. 10.18632/oncotarget.24423 29568380 PMC5862601

[B18] Hradsky J. Bernstein H. -G. Marunde M. Mikhaylova M. Kreutz M. R. (2015). Alternative splicing, expression and cellular localization of Calneuron-1 in the rat and human brain. *J. Histochem. Cytochem.* 63 793–804. 10.1369/0022155415595841 26116628 PMC4823805

[B19] Hradsky J. Mikhaylova M. Karpova A. Kreutz M. R. Zuschratter W. (2013). Super-resolution microscopy of the neuronal calcium-binding proteins Calneuron-1 and Caldendrin. *Methods Mol. Biol.* 963 147–69. 10.1007/978-1-62703-230-8_10 23296610

[B20] Iqbal K. Liu F. Gong C.-X. Grundke-Iqbal I. (2010). Tau in Alzheimer disease and related tauopathies. *Curr. Alzheimer Res.* 7 656–664. 10.2174/156720510793611592 20678074 PMC3090074

[B21] Jo J. Heon S. Kim M. J. Son G. H. Park Y. Henley J. M. (2008). Metabotropic glutamate receptor-mediated LTD involves two interacting Ca^2+^Sensors, NCS-1 and PICK1. *Neuron* 60 1095–1111. 10.1016/j.neuron.2008.10.050 19109914 PMC3310905

[B22] Laurie D. J. Bartke I. Schoepfer R. Naujoks K. Seeburg P. H. (1997). Regional, developmental and interspecies expression of the four NMDAR2 subunits, examined using monoclonal antibodies. *Mol. Brain Res.* 51 23–32. 10.1016/S0169-328X(97)00206-4 9427503

[B23] Lian L. Y. Pandalaneni S. R. Patel P. McCue H. V Haynes L. P. Burgoyne R. D. (2011). Characterisation of the interaction of the c-terminus of the dopamine d2 receptor with neuronal calcium sensor-1. *PLoS One* 6:e27779. 10.1371/journal.pone.0027779 22114693 PMC3218054

[B24] Masuda-Suzukake M. Nonaka T. Hosokawa M. Kubo M. Shimozawa A. Akiyama H. (2014). Pathological alpha-synuclein propagates through neural networks. *Acta Neuropathol. Commun.* 2:88. 10.1186/s40478-014-0088-8 25095794 PMC4147188

[B25] Matsuda L. A. Lolait S. J. Brownstein M. J. Young A. C. Bonner T. I. (1990). Structure of a cannabinoid receptor and functional expression of the cloned cDNA. *Nature* 346 561–564. 10.1038/346561a0 2165569

[B26] McCue H. V. Haynes L. P. Burgoyne R. D. (2010). The diversity of calcium sensor proteins in the regulation of neuronal function. *Cold Spring Harb. Perspect. Biol.* 2 a004085–a004085. 10.1101/cshperspect.a004085 20668007 PMC2908765

[B27] Medina M. Avila J. (2014). The role of extracellular Tau in the spreading of neurofibrillary pathology. *Front. Cell. Neurosci.* 8:113 10.3389/fncel.2014.00113 PMC4005959 24795568

[B28] Mikhaylova M. Bär J. Bommel B. van Schätzle P. YuanXiang P. A. Raman R. (2018). Caldendrin directly couples postsynaptic calcium signals to actin remodeling in dendritic spines. *Neuron* 97 1110.e14–1125.e14. 10.1016/j.neuron.2018.01.046 29478916

[B29] Mikhaylova M. Hradsky J. Kreutz M. R. (2011). Between promiscuity and specificity: novel roles of EF-hand calcium sensors in neuronal Ca2+ signalling. *J. Neurochem.* 118 695–713. 10.1111/j.1471-4159.2011.07372.x 21722133

[B30] Mikhaylova M. Reddy P. P. Munsch T. Landgraf P. Suman S. K. Smalla K.-H. (2009). Calneurons provide a calcium threshold for trans-Golgi network to plasma membrane trafficking. *Proc. Natl. Acad. Sci. U.S.A.* 106 9093–9098. 10.1073/pnas.0903001106 19458041 PMC2690001

[B31] Mikhaylova M. Sharma Y. Reissner C. Nagel F. Aravind P. Rajini B. (2006). Neuronal Ca2+ signaling via caldendrin and calneurons. *Biochim. Biophys. Acta – Mol. Cell Res.* 1763 1229–1237. 10.1016/j.bbamcr.2006.08.047 17055077

[B32] Mishizen-Eberz A. J. Rissman R. A. Carter T. L. Ikonomovic M. D. Wolfe B. B. Armstrong D. M. (2004). Biochemical and molecular studies of NMDA receptor subunits NR1/2A/2B in hippocampal subregions throughout progression of Alzheimer’s disease pathology. *Neurobiol. Dis.* 15 80–92. 10.1016/j.nbd.2003.09.016 14751773

[B33] Monyer H. Burnashev N. Laurie D. J. Sakmann B. Seeburg P. H. (1994). Developmental and regional expression in the rat brain and functional properties of four NMDA receptors. *Neuron* 12 529–540. 10.1016/0896-6273(94)90210-0 7512349

[B34] Mucke L. Masliah E. Yu G. Q. Mallory M. Rockenstein E. M. Tatsuno G. (2000). High-level neuronal expression of abeta 1-42 in wild-type human amyloid protein precursor transgenic mice: synaptotoxicity without plaque formation. *J. Neurosci.* 20 4050–4058. 10.1523/JNEUROSCI.20-11-04050.2000 10818140 PMC6772621

[B35] Mudher A. Colin M. Dujardin S. Medina M. Dewachter I. Naini S. M. A. (2017). What is the evidence that tau pathology spreads through prion-like propagation? *Acta Neuropathol. Commun.* 5:99. 10.1186/s40478-017-0488-7 29258615 PMC5735872

[B36] Nakamura T. Y. Nakao S. Nakajo Y. Takahashi J. C. Wakabayashi S. Yanamoto H. (2017). Possible signaling pathways mediating neuronal calcium sensor-1-dependent spatial learning and memory in Mice. *PLoS One* 12:e0170829. 10.1371/journal.pone.0170829 28122057 PMC5266288

[B37] Navarro G. Aguinaga D. Moreno E. Hradsky J. Reddy P. P. Cortés A. (2014). Intracellular calcium levels determine differential modulation of allosteric interactions within G protein-coupled receptor heteromers. *Chem. Biol.* 21 1546–1566. 10.1016/j.chembiol.2014.10.004 25457181 PMC9875831

[B38] Navarro G. Aymerich M. S. Marcellino D. Cortés A. Casadó V. Mallol J. (2009). Interactions between calmodulin, adenosine A2A, and dopamine D2receptors. *J. Biol. Chem.* 284 28058–28068. 10.1074/jbc.M109.034231 19632986 PMC2788857

[B39] Navarro G. Borroto-Escuela D. Angelats E. Etayo Í. Reyes-Resina I. Pulido-Salgado M. (2018). Receptor-heteromer mediated regulation of endocannabinoid signaling in activated microglia. Relevance for Alzheimer’s disease and levo-dopa-induced dyskinesia. *Brain. Behav. Immun.* 67 139–151. 10.1016/j.bbi.2017.08.015 28843453

[B40] Navarro G. Franco N. Martínez-Pinilla E. Franco R. (2017). The epigenetic cytocrin pathway to the nucleus. Epigenetic factors, epigenetic mediators, and epigenetic traits. A biochemist perspective. *Front. Genet.* 8:179. 10.3389/fgene.2017.00179 29230234 PMC5711780

[B41] Navarro G. Hradsky J. Lluís C. Casadó V. McCormick P. J. Kreutz M. R. (2012a). NCS-1 associates with adenosine A(2A) receptors and modulates receptor function. *Front. Mol. Neurosci.* 5:53. 10.3389/fnmol.2012.00053 22529776 PMC3328853

[B42] Navarro G. Hradsky J. Lluís C. Casadó V. McCormick P. J. Kreutz M. R. (2012b). NCS-1 associates with adenosine A2A receptors and modulates receptor function. *Front. Mol. Neurosci.* 5:53. 10.3389/fnmol.2012.00053 22529776 PMC3328853

[B43] Newell E. Exo J. Verrier J. Jackson T. Gillespie D. Janesko-Feldman K (2015). 2′,3′-cAMP, 3′-AMP, 2′-AMP and adenosine inhibit TNF-α and CXCL10 production from activated primary murine microglia via A2A receptors. *Brain Res.* 1594 27–35. 10.1016/j.brainres.2014.10.059 25451117 PMC4262711

[B44] Pacheco R. Martinez-Navio J. M. M. Lejeune M. Climent N. Oliva H. Gatell J. M. M. (2005). CD26, adenosine deaminase, and adenosine receptors mediate costimulatory signals in the immunological synapse. *Proc. Natl. Acad. Sci. U.S.A.* 102 9583–9588. 10.1073/pnas.0501050102 15983379 PMC1172240

[B45] Pankratov Y. Lalo U. (2014). Calcium permeability of ligand-gated Ca2+ channels. *Eur. J. Pharmacol.* 739 60–73. 10.1016/j.ejphar.2013.11.017 24291105

[B46] Paoletti P. Bellone C. Zhou Q. (2013). NMDA receptor subunit diversity: impact on receptor properties, synaptic plasticity and disease. *Nat. Rev. Neurosci.* 14 383–400. 10.1038/nrn3504 23686171

[B47] Paterlini M. Valerio A. Baruzzi F. Memo M. Spano P. (1998). Opposing regulation of tau protein levels by ionotropic and metabotropic glutamate receptors in human NT2 neurons. *Neurosci. Lett.* 243 77–80. 10.1016/S0304-3940(98)00087-1 9535117

[B48] Pérez M. Valpuesta J. M. Medina M. Montejo de Garcini E. Avila J. (2002). Polymerization of τ into filaments in the presence of heparin: the minimal sequence required for τ - τ interaction. *J. Neurochem.* 67 1183–1190. 10.1046/j.1471-4159.1996.67031183.x 8752125

[B49] Raghuram V. Sharma Y. Kreutz M. R. (2012). Ca2+ sensor proteins in dendritic spines: a race for Ca2+. *Front. Mol. Neurosci.* 5:61. 10.3389/fnmol.2012.00061 22586368 PMC3347464

[B50] Rönicke R. Mikhaylova M. Rönicke S. Meinhardt J. Schröder U. H. Fändrich M. (2011). Early neuronal dysfunction by amyloid β oligomers depends on activation of NR2B-containing NMDA receptors. *Neurobiol. Aging* 32 2219–28. 10.1016/j.neurobiolaging.2010.01.011 20133015

[B51] Ruiz M. A. Escriche M. Lluis C. Franco R. Martín M. Andrés A. (2000). Adenosine A(1) receptor in cultured neurons from rat cerebral cortex: colocalization with adenosine deaminase. *J. Neurochem.* 75 656–664. 10.1046/j.1471-4159.2000.0750656.x 10899940

[B52] Seidenbecher C. I. Langnaese K. Sanmartí-Vila L. Boeckers T. M. Smalla K. H. Sabel B. A. (1998). Caldendrin, a novel neuronal calcium-binding protein confined to the somato-dendritic compartment. *J. Biol. Chem.* 273 21324–21331. 10.1074/jbc.273.33.21324 9694893

[B53] Szeto J. Y. Y. Lewis S. J. G. (2016). Current treatment options for Alzheimer’s disease and Parkinson’s disease dementia. *Curr. Neuropharmacol.* 14 326–38. 10.2174/1570159x14666151208112754 26644155 PMC4876589

[B54] Tarutani A. Arai T. Murayama S. Hisanaga S. Hasegawa M. (2018). Potent prion-like behaviors of pathogenic α-synuclein and evaluation of inactivation methods. *Acta Neuropathol. Commun.* 6:29. 10.1186/s40478-018-0532-2 29669601 PMC5907316

[B55] Tarutani A. Suzuki G. Shimozawa A. Nonaka T. Akiyama H. Hisanaga S. I. (2016). The effect of fragmented pathogenic α-synuclein seeds on prion-like propagation. *J. Biol. Chem.* 291 18675–18688. 10.1074/jbc.M116.734707 27382062 PMC5009244

[B56] Ułas J. Cotman C. W. (1997). Decreased expression of N-methyl-D-aspartate receptor 1 messenger RNA in select regions of Alzheimer brain. *Neuroscience* 79 973–982. 10.1016/S0306-4522(97)00023-7 9219960

[B57] Wakabayashi K. Narisawa-Saito M. Iwakura Y. Arai T. Ikeda K. Takahashi H. (1999). Phenotypic down-regulation of glutamate receptor subunit GluR1 in Alzheimer’s disease. *Neurobiol. Aging* 20 287–295. 10.1016/S0197-4580(99)00035-4 10588576

[B58] Wang J. Q. Fibuch E. E. Mao L. (2007). Regulation of mitogen-activated protein kinases by glutamate receptors. *J. Neurochem.* 100 1–11. 10.1111/j.1471-4159.2006.04208.x 17018022

[B59] Watanabe M. Inoue Y. Sakimura K. Mishina M. (1993). Distinct distributions of five N-methyl-D-aspartate receptor channel subunit mRNAs in the forebrain. *J. Comp. Neurol.* 338 377–90. 10.1002/cne.903380305 8113446

[B60] Woodhall G. Evans D. I. Cunningham M. O. Jones R. S. (2001). NR2B-containing NMDA autoreceptors at synapses on entorhinal cortical neurons. *J. Neurophysiol.* 86 1644–51. 10.1152/jn.2001.86.4.1644 11600627

