# Supplementary figures and images for: N-Methyl-D-Aspartate Receptor Link to the MAP Kinase Pathway in Cortical and Hippocampal Neurons and Microglia Is Dependent on Calcium Sensors and Is Blocked by α-Synuclein, Tau, and Phospho-Tau in Non-transgenic and Transgenic APPSw,Ind Mice
Source: Front Mol Neurosci. 2018 Aug 28;11:273. doi: 10.3389/fnmol.2018.00273 (PMC6127644; doi:10.3389/fnmol.2018.00273)

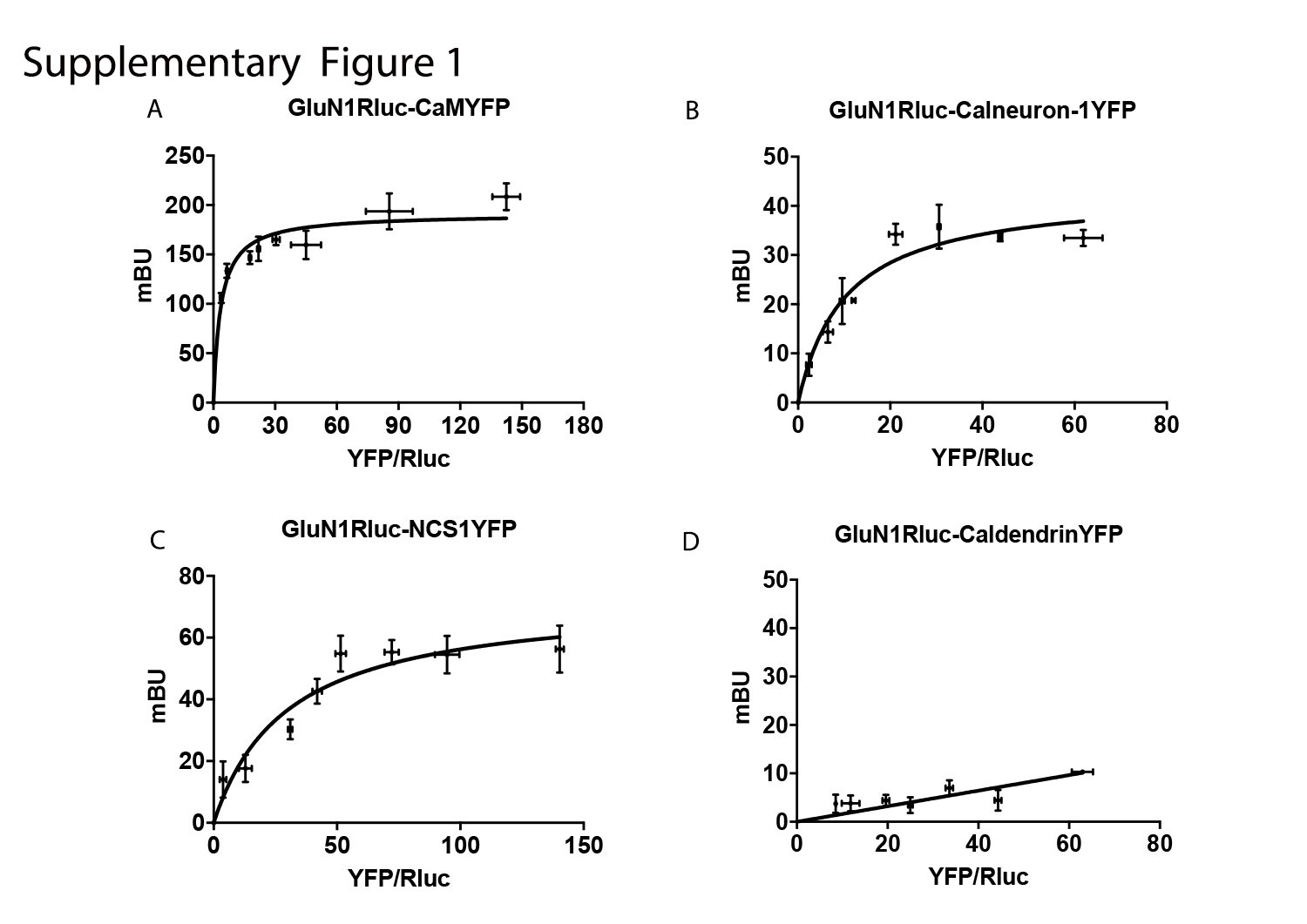

Supplement: FIGURE S1 — The N1 subunit of NMDAR receptor may interact with calmodulin, calneuron-1 and NCS1. (A–D) BRET saturation experiments in HEK-293T cells transfected with cDNAs for GluN1Rluc (0.3 μg), and increasing amounts of cDNA for CaMYFP (0.05–0.4 μg) (A), calneuron-1YFP (0.05–1 μg) (B), NCS1YFP (0.05–0.6 μg) (C), or caldendrin YFP (0.05–1 μg) (D). Values are the mean ± SEM (n = 6). [file Image_1.TIF]

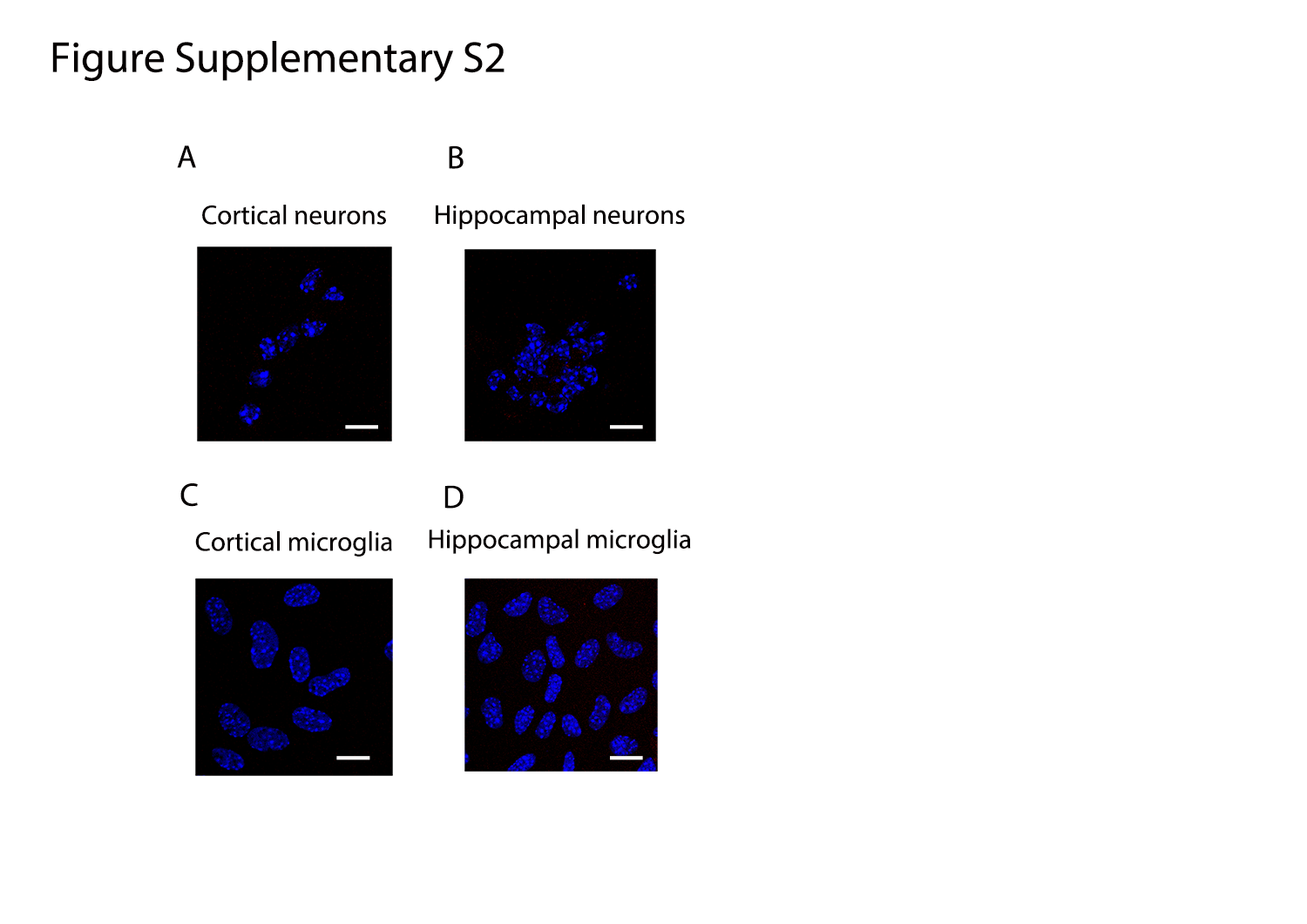

Supplement: FIGURE S2 — Proximity ligation assay (PLA) negative controls for PLAs in primary cultures of neurons and microglia. PLAs were performed in cortical (A neurons, C microglia) and hippocampal (B neurons, D microglia) cells incubated with the anti-CaM antibody but omitting the anti-NMDAR antibody. Confocal microscopy images are shown (superimposed sections) in which heteromers appear as red clusters (in neurons or microglia). Scale bars = 10 μm (neurons) and 20 μm (microglia). In all cases, cell nuclei were stained with Hoechst (blue). [file Image_2.TIF]

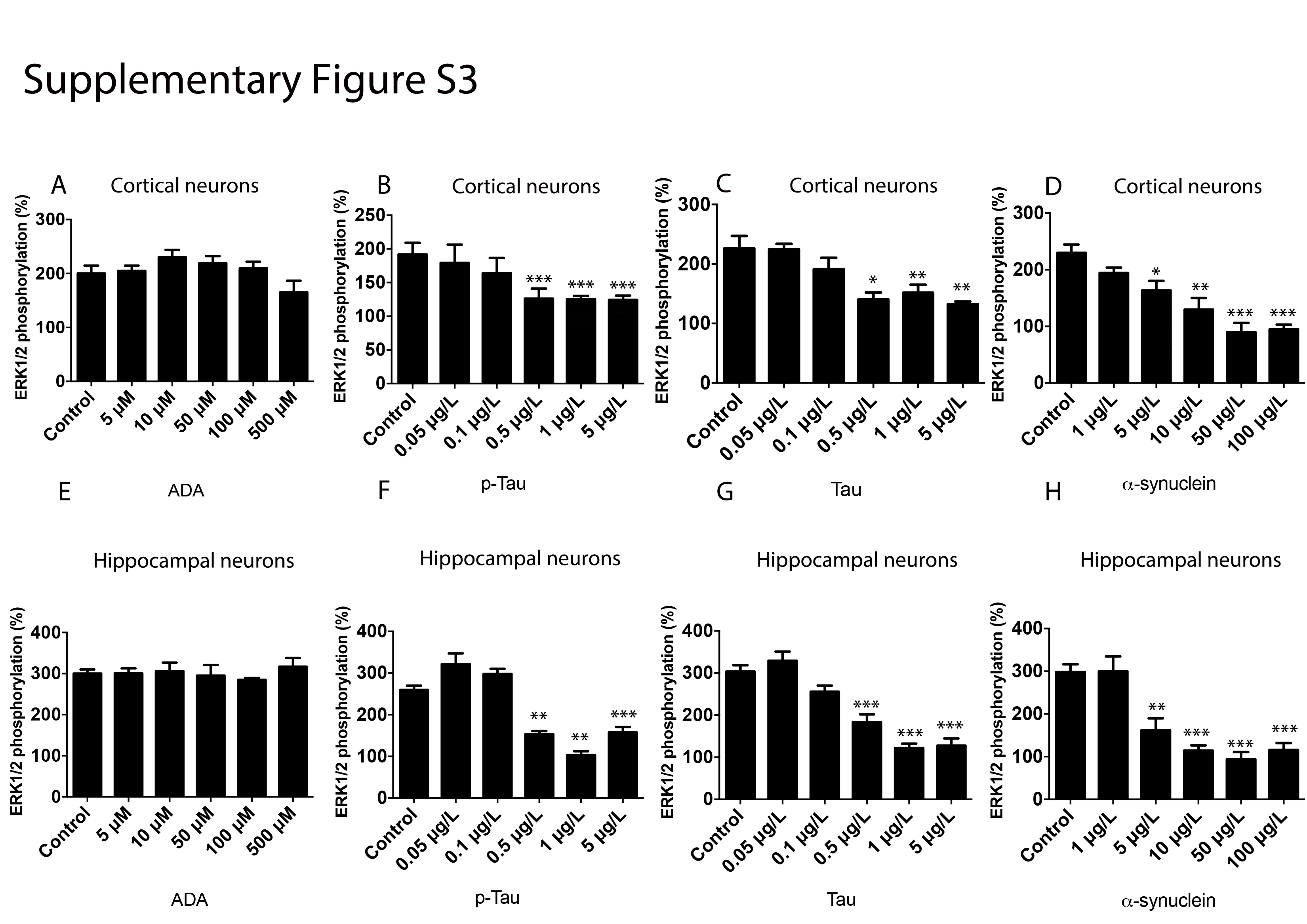

Supplement: FIGURE S3 — Tau, p-Tau, and α-synuclein dose–response curves in neuronal primary cultures. (A–H) MAPK phosphorylation levels were analyzed in primary cultures of cortical (A–D), or hippocampal neurons (E–H). Assays were performed in cells treated with increasing concentrations of adenosine deaminase (ADA) (5–500 μM). (A,E) Tau (0.05–5 μg/L) (B,F), p-Tau (0.05–5 μg/L) (C,G), or α-synuclein (1 μM–100 μg/L) (D,H) for 2 h prior to 15 μM NMDA stimulation. Values are the mean ± SEM (n = 6). Significant differences over NMDA treatment (control condition) (∗p < 0.05, ∗∗p < 0.01, and ∗∗∗p < 0.005) were calculated by one-way ANOVA and Bonferroni post hoc test. [file Image_3.TIF]

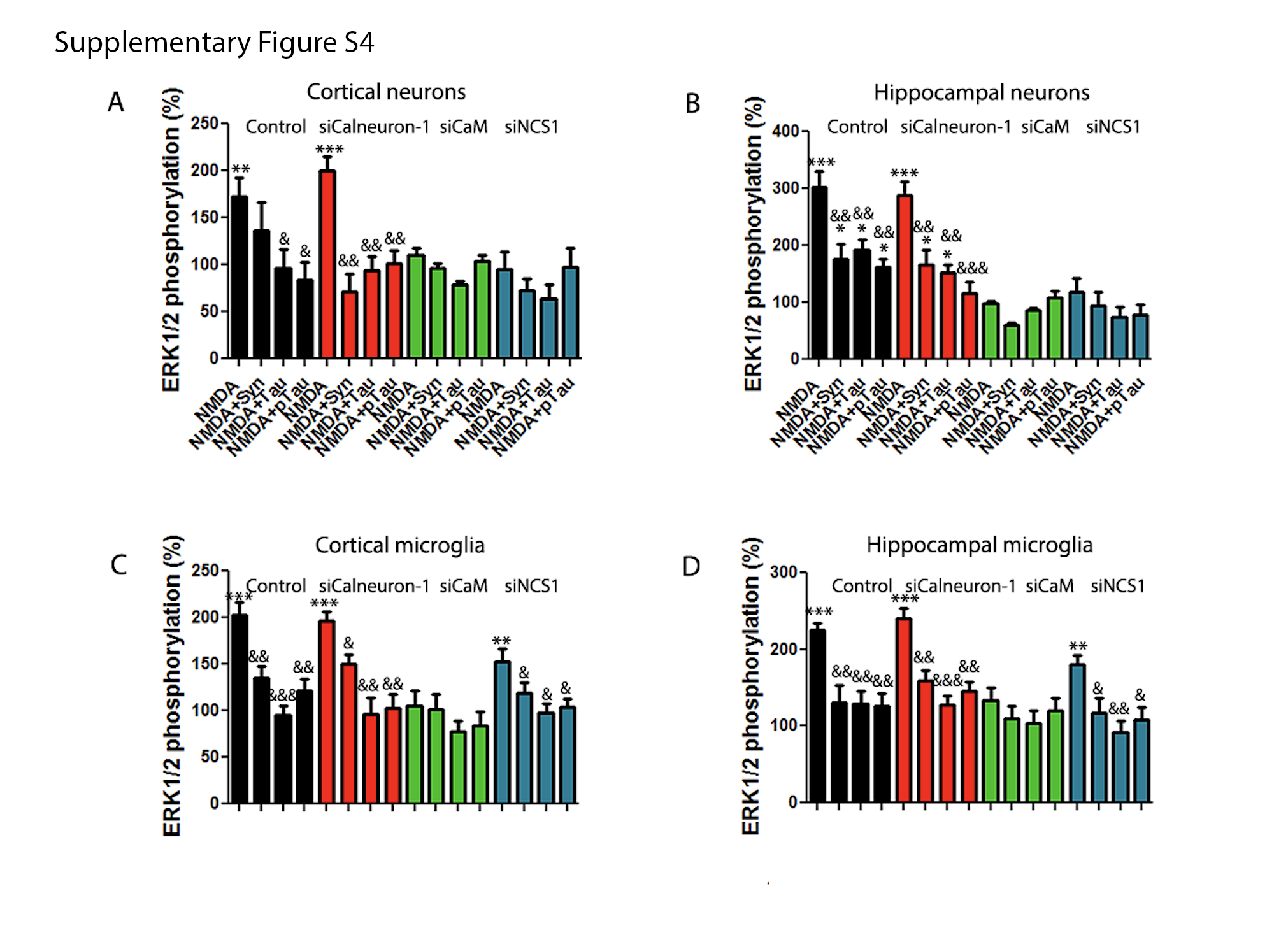

Supplement: FIGURE S4 — Chronic treatment with Tau, p-Tau, or α-synuclein inhibits NMDAR-mediated signaling in primary cultures. (A–D) MAPK phosphorylation levels were analyzed after stimulating primary cultures of cortical (A), or hippocampal neurons (B), or of cortex (C), or hippocampal microglia (D). Experiments were performed in cells transfected or not (black bars) with siRNA to silence calneuron-1 (red bars), CaM (green bars), or NCS1 (blue bars) expression. Assays were performed in cells treated with α-synuclein, Tau or p-Tau for 7 days prior to 15 μM NMDA addition. Labels in X axis are equal in all bar graphs. Values are the mean ± SEM (n = 10). Significant differences over non-treated cells (∗∗p < 0.01, ∗∗∗p < 0.005) or over NMDA treatment (&p < 0.05, &&p < 0.01, and &&&p < 0.001) were calculated by one-way ANOVA and Bonferroni post hoc test. [file Image_4.TIF]

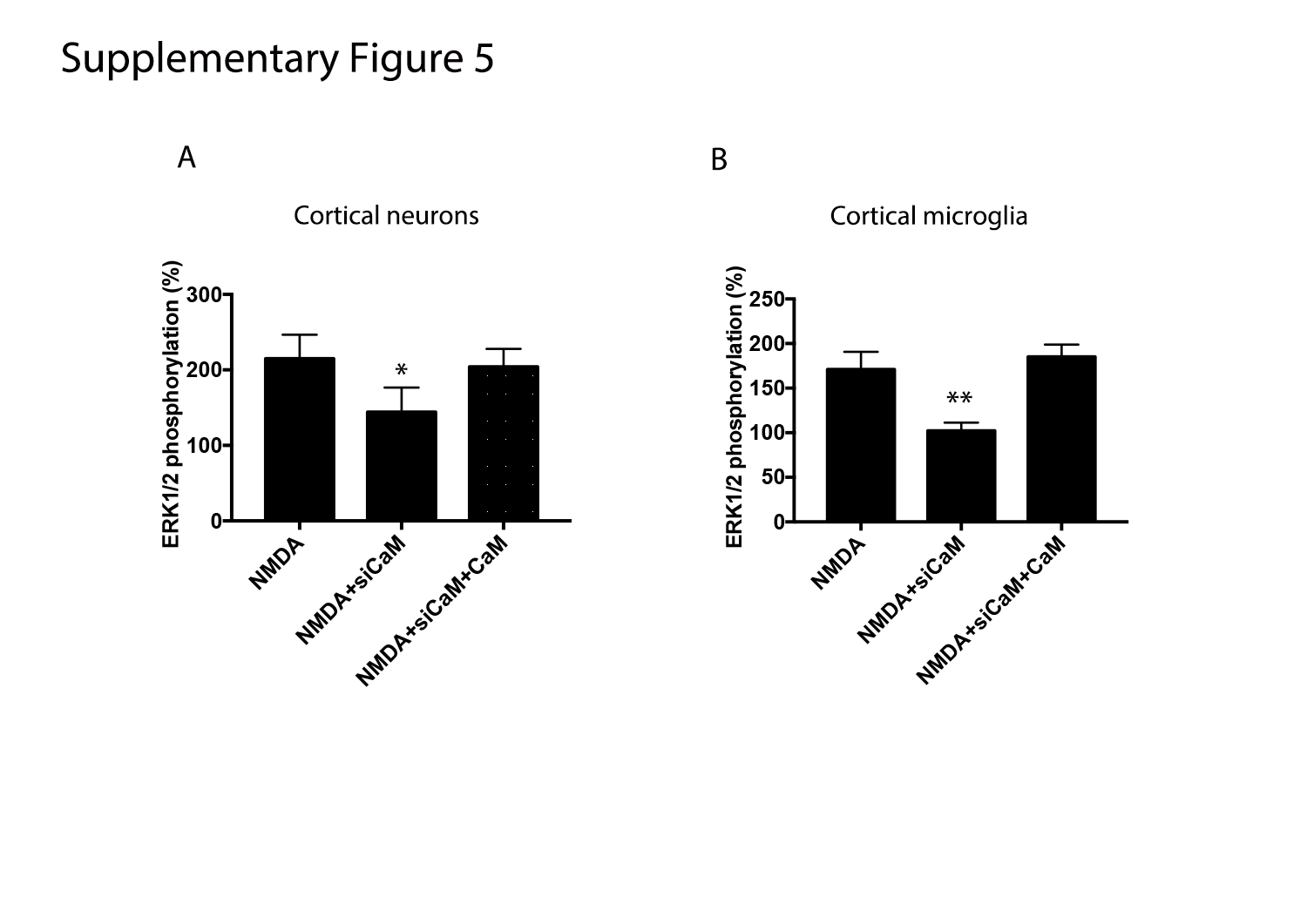

Supplement: FIGURE S5 — Rescue of (endogenous) CaM silencing upon transfection with the cDNA for CaM. (A,B) MAPK phosphorylation levels were analyzed after stimulating primary cultures of cortical neurons (A) or cortical microglia (B) with 15 μM NMDA. Experiments were performed in cells transfected or not with siRNA to silence CaM; 24 h later, cells were transfected with cDNA for CaM or with the (empty) pcDNA3.1 vector. Experiments in untransfected (left), in siRNA (center), and in siRNA plus CaM (right) cells were preformed simultaneously. Data are the mean ± SEM (n = 5). One-way ANOVA followed by Bonferroni’s multiple comparison post hoc test were used for statistics analysis (∗p < 0.05, ∗∗p < 0.01 versus NMDA treatment). [file Image_5.TIF]
